# Posterior-stabilized total knee arthroplasty kinematics and joint laxity: A hybrid biomechanical study

**DOI:** 10.1186/s42836-022-00153-4

**Published:** 2022-12-15

**Authors:** Allan R. Sekeitto, Jance G. McGale, Liam A. Montgomery, Edward M. Vasarhelyi, Ryan Willing, Brent A. Lanting

**Affiliations:** 1 London Health Sciences Centre, 339 Windermere Rd, London, ON N6A 5A5 Canada; 2 Department of Mechanical & Materials Engineering, University of Western Ontario, 1151 Richmond Street N., London, ON N6A 5B9 Canada

**Keywords:** Posterior-stabilized total knee arthroplasty (PS-TKA), Kinematics, Joint laxity, Joint motion simulator, Virtual ligament model

## Abstract

**Background:**

Posterior-stabilized (PS)-total knee arthroplasty (TKA) arose as an alternative to cruciate-retaining (CR)-TKA in the 1970s. Since then, it has become a popularly utilized TKA design with outcomes comparable to CR-TKA. The post-cam mechanism is unique to PS-TKA as it substitutes the function of the posterior cruciate ligament (PCL). The study aimed to understand the kinematic and laxity changes in PS-TKA with under- and overstuffing of the tibiofemoral joint space with the polyethylene (PE) insert.

**Methods:**

This study employed a hybrid computational-experimental joint motion simulation on a VIVO 6 degrees of freedom (6-DoF) joint motion simulator (AMTI, Watertown, MA, USA). Physical prototypes of a virtually-performed TKA in mechanical alignment (MA) and kinematic alignment (KA) based on cadaveric CT scans and a virtual ligament model were utilized. The reference, understuffed (down 2 mm) and overstuffed (up 2 mm) joint spaces were simulated, neutral flexion and laxity testing loads and motions were performed for each configuration.

**Results:**

The PE insert thickness influenced post-cam engagement, which occurred after 60º in the overstuffed configurations, after 60º–75º in the reference configurations and after 75º in the understuffed configurations. The understuffed configurations, compared to the reference configurations, resulted in a mean 2.0º (28%) and 2.0º (31%) increase in the coronal laxity in MA and KA respectively. The overstuffed configurations, compared to the reference configuration, resulted in an increase in the mean joint compressive forces (JCFs) by 73 N (61%) and 77 N (62%) in MA and KA models, respectively.

**Conclusions:**

The under- and overstuffing in PS-TKA alter the kinematics with variable effects. Understuffing decreases the stability, JCFs and inverse with overstuffing. Subtle changes in the PE insert thickness alter the post-cam mechanics.

## Introduction

Posterior-stabilized (PS)-total knee arthroplasty (TKA), arose as an alternative to cruciate-retaining (CR)-TKA in the 1970s. Since then, it has become a popularly utilized TKA design with outcomes comparable to CR-TKA [1]. The post-cam mechanism is unique to PS-TKA as it substitutes the degenerative posterior cruciate ligament (PCL), thereby preventing posterior tibial subluxation, facilitating femoral rollback and improving range of motion (ROM) [2, 3]. There are reported design variations in the post-cam mechanism [3, 4]. The principle of the design is a post which is located on the polyethylene (PE) insert and the cam is on the femoral component, as the knee flexes they engage facilitating the femoral rollback [5].

Whilst there has been great success in TKA, a subset of patients remain dissatisfied with their results [6, 7]. The primary goal of TKA is to provide a painless stable knee and to restore joint function in patients with advanced osteoarthritis. The knee prosthesis should be performed in a manner to provide a maximal ROM while providing stability [8]. With TKA, pain and instability are common indications for revision [9–11]. A literature review has shown that postoperative stiffness is a relatively common outcome, accounting for 4–16% [12]. This highlights the challenge of providing a stable and painless TKA.

Recent advances in TKA include increasing technology in the operating room [13]. However, the judgement as to what PE insert size to use—what is too tight, too loose, symmetric tightness and soft tissue balancing remain unclear. Manual techniques utilized over and above traditional balancing include spacer blocks, lamina spreaders, tensiometers and stress tests are common practice but are subjective [14]. Technologies such as intra-component force sensing devices were developed to aid the intraoperative surgeon decision-making [15]. The early data indicate this technology may improve outcomes in TKA [16, 17]. However, it remains unclear what the correct answer is, as there is no consensus as to what defines the balanced knee.

The advances in joint motion simulator technology have enabled these machines to analyze TKA mechanics while simulating different alignments and soft tissue balancing, by changing the properties of parametric virtual ligaments [18]. We hypothesized that either under- or overstuffing with the PE insert would affect knee kinematics. The objective of this study was to determine the differences in kinematics with PE thickness in PS-TKA using a joint motion simulator machine linked to a virtual ligament model.

## Methods

**Fig. 1:**
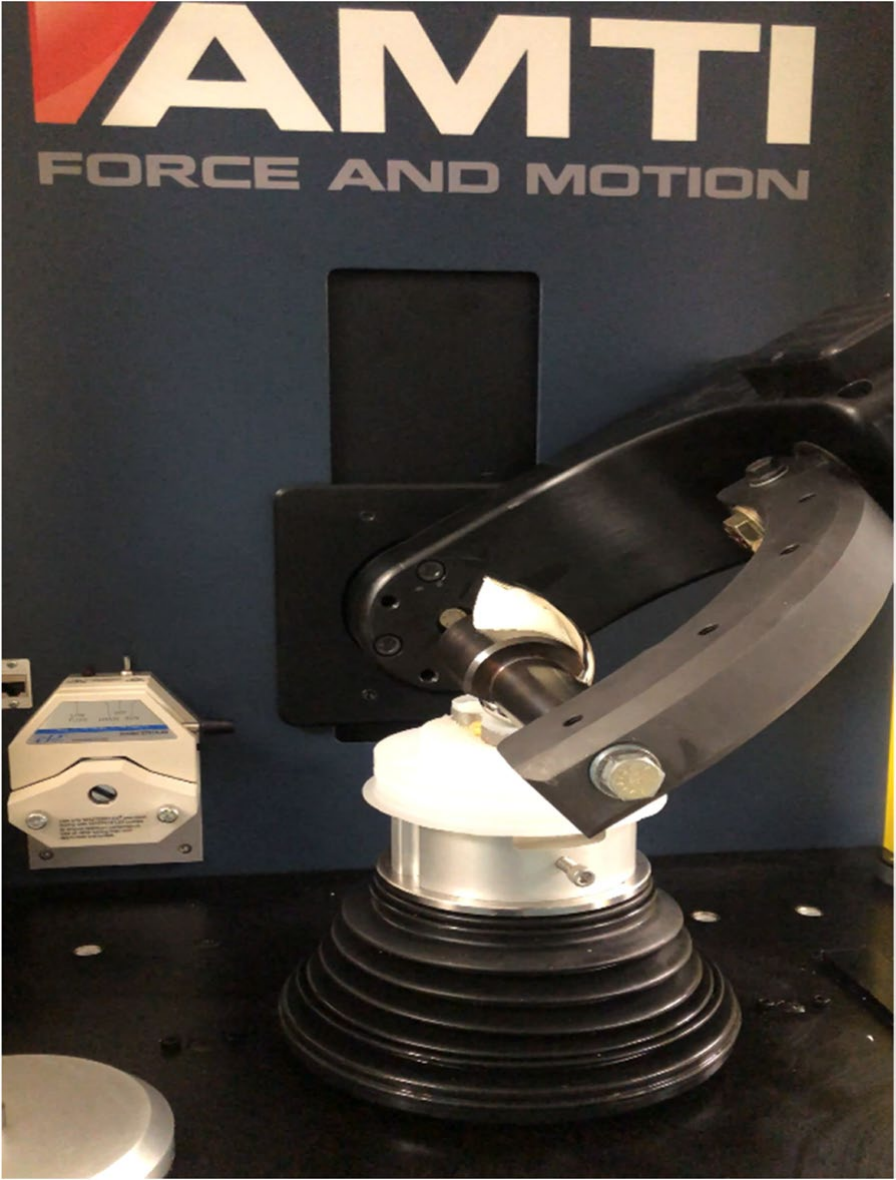
VIVO 6 degrees of freedom joint motion simulator (AMTI, Watertown, MA, USA)

This study employed a hybrid computational-experimental joint motion simulation on a VIVO 6 degrees of freedom (6-DoF) joint motion simulator (AMTI, Watertown, MA, USA) (Fig. 1). These simulations measure the kinematics of physical implant components in response to applied loads, but with forces imposed as a result of simulated stretching of surrounding "virtual ligaments". These simulated one-dimensional point-to-point springs are virtually installed around the implant components and will become tensioned or slack in response to insertion kinematics (insertion coordinates defined relative to the implant components), and the forces they generate will contribute to joint kinematics and stability. The virtual ligaments employed in the current study were designed to replicate the relative insertion coordinates, tensioning and stiffness of real ligaments around a TKA, using the following procedure.

### Virtual ligament design

The distal femur and proximal tibia were reconstructed from a CT scan of a single cadaver knee in neutral extension using 3D Slicer [19] and exported as stereolithography (.stl) files. These 3D surface models were imported into the commercially available CAD software, SolidWorks 2020® (Dassault Systèmes SolidWorks Corporation, Waltham, MA, USA). In SolidWorks, this native knee geometry was used to identify relevant ligament insertions based on established bony landmarks and previous literature [20–27]. The femoral and tibial insertions of the superficial medial collateral ligament (sMCL) and lateral collateral ligament (LCL) were each identified and would serve as connection points for the single-bundle virtual ligaments. The anterior and posterior cruciate ligaments and deep MCL were not represented as they are routinely released in PS-TKA procedures as part of the soft tissue dissection required for exposure or bony resection. The insertion-to-insertion distance of each ligament was noted. Acknowledging that ligaments may be slack or stretched when in neutral extension, these distances could not be used to define the true resting length (unstretched or “slack” length) of each ligament. The slack length, however, was estimated based on literature-derived intact knee ligament reference strains and the insertion-to-insertion distance in extension [28–30].

Surface models (.stl files) of a tibial and femoral Triathlon^®^ (Stryker Corp., Mahwah, NJ, USA) TKA components were imported into the CAD software. A size 5 tibial tray with a 9-mm thick PS PE insert component was used, in combination with a size 5 femoral component. Virtual TKA was performed with mechanical alignment (MA) and kinematic alignment (KA). In MA the distal femur bone model resection was perpendicular to the femur's mechanical axis, and for the proximal tibia resection was perpendicular to the tibia's mechanical axis. The femoral component was aligned with the approximated trans-epicondylar axis; this was determined by externally rotating 3° from the posterior condylar axis. In KA the distal femur resection was made 3° valgus to the femur's mechanical axis and the proximal tibial resection was made 3° varus to the tibia's mechanical axis. The femoral rotation was set to align parallel with the posterior condylar axis. In both alignments, the posterior tibial slope was a 3° resection.

The implant components were aligned to the cut surfaces of the femur and tibia, and then the entire tibia (plus the tibial component) was translated and rotated such that the femoral and tibial components were neutrally positioned and aligned (in extension, with the femoral condyles dwelling at the deepest point in the PE dishes). The coordinates of the ligament femoral and tibial insertions were measured with respect to the femoral component, as were their new lengths which changed after TKA. Along with the ligament stiffness, these data were sufficient to define virtual ligaments around the TKA.

### Joint motion simulator

Real physical prototypes of the same implant components were mounted onto the VIVO. The femoral component was mounted to the mounting axle with polymethyl methacrylate cement (Bosworth Fastray; Keystone Industries GmbH, Singen, Germany) and the tibial baseplate component was anchored into the tibial fixture using dental model stone (Modern Materials Golden Denstone Labstone; Modern Materials, Kulzer GmbH, Hanau, Germany). We used polydimethylsiloxane (silicone)-based lubricant (HAS0001-OS, Horizon Fitness, Cottage Grove, WI, USA) as an articulation lubricant and applied it consistently throughout the duration of the experiment. The VIVO was used to apply loads and motions representative of neutral flexion and laxity testing, and the resulting kinematics (measured outcome) were sensitive to the implant component geometries, alignments and virtual ligament properties. This in vitro technique of measuring motions and kinematics with simulated virtual ligaments has been previously described [18].

### Joint space

Three different joint spaces were simulated:

1. Reference joint space: The 9-mm PE insert was utiliszed. This created our reference joint space relative to our virtual ligament model.
2. Understuffed joint space: The 9-mm PE insert was undersized by 2 mm. This was simulated by the VIVO and resulted in an understuffed joint space.
3. Overstuffed joint space: The 9-mm PE insert was oversized by 2 mm. This was simulated by the VIVO and resulted in an overstuffed joint space.

### Input loads and motions

Motions were simulated as follows:

(i) Neutral Flexion Kinematics: A 10 N compressive force applied parallel to the long axis of the tibia and passing through the centre of the joint, the femur was flexed to 90° and extended back to 0° at a rate of 25 s/cycle. All other degrees of freedom were unconstrained (set to maintain 0 N or 0 Nm of load). Four flexion/extension cycles were simulated; resulting 6-DoFs joint kinematics, net ligament forces and individual ligament tensions were recorded during the 3rd and 4th iterations.
(ii) Posterior Laxity: A 10 N compressive force applied parallel to the long axis of the tibia and passing through the centre of the joint, the joint was flexed from 0° to 90° in 15° increments. At each fixed flexion angle, a 100 N posterior-directed force was applied to the tibia, causing its relative posterior displacement. This posterior displacement was limited by the combined contributions of the concave congruency of the condyles and tensioning of the virtual ligaments. All other degrees of freedom were unconstrained. After testing at each flexion angle, the joint returned to 0° and the entire process was repeated, for a total of four iterations. Data were recorded during the 3rd and 4th iterations. Recorded data included the posterior displacement of the tibia (relative to the corresponding neutral flexion kinematics at the same flexion angle), net ligament forces and individual ligament tensions.
(iii) Varus/Valgus (VV) Laxity: VV laxity testing was accomplished using a similar technique as described for (ii), but by applying a 10 Nm varus or valgus joint torque instead of a 100 N posterior force. Recorded data included the varus/valgus angulation (relative to the corresponding neutral flexion kinematics at the same flexion angle), net ligament forces and individual ligament tensions.

### Data analysis and statistics

Recorded data were smoothed using a low‐pass Butterworth filter followed by a spline interpolation function in Matlab (The MathWorks, Natick, MA, USA), and then down‐sampled to only include data at 15° intervals of flexion and only during the flexion phase of the complete flexion/extension motion in the laxity testing. During the motion testing, the joint motion was sampled throughout the cycle we extracted the anteroposterior (AP), internal/external rotation (IE) and VV kinematic data in each of the 6-DoFs. We also collected posterior, varus and valgus motion limits in each of the 6-DoFs at these limits. The smoothed and processed data were used for statistical analysis. Descriptive statistics were used to compare the results between each joint space configuration and alignment. All statistical analyses were completed in Microsoft® Excel (v.16.45).

## Results

### Neutral flexion

**Fig. 2:**
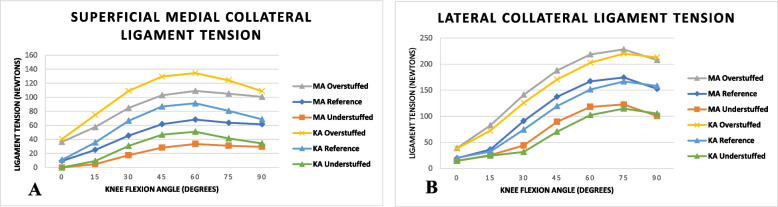
Graphs demonstrating the relationship between ligament tension and polyethylene insert thickness. **A** Superficial medial collateral ligament, **B** Lateral collateral ligament

The PE insert thickness influenced the ligament tension during neutral flexion (shown in Fig. 2)*.* The means values across the entire neutral flexion arc (0°–90°) for the 2-mm increase (overstuffed) or decrease (understuffed) in PE insert thickness, the sMCL tension, increased or decreased by a mean 32 N (105%) and 36 N (85%) in MA and KA respectively. For the 2-mm increase (overstuffed) or decrease (understuffed) PE inserts thickness, the LCL tension increased or decreased by a mean of 42 N (47%) and 41 N (50%) in MA and KA respectively. The adduction moment observed in the configurations resulted in the LCL tension being higher than the sMCL tension.

**Fig. 3:**
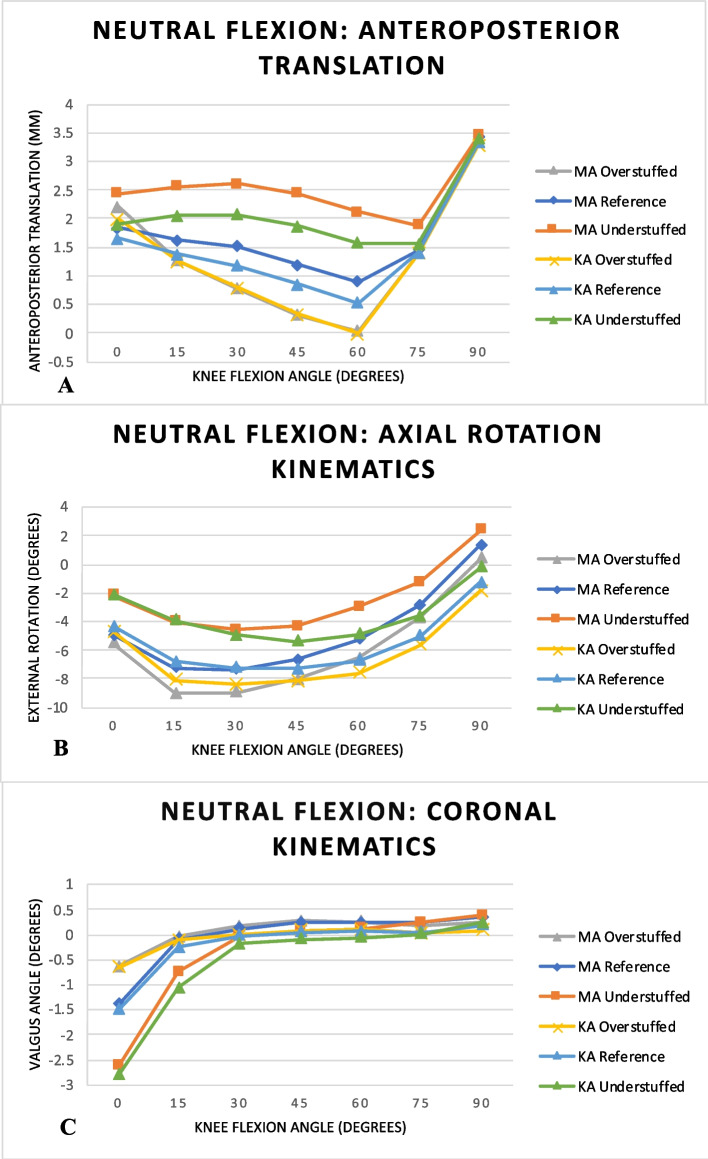
Graphs demonstrating the neutral flexion kinematics. **A** Anteroposterior, **B** Axial Rotation, **C** Varus/valgus

The neutral flexion AP translation kinematics are shown in Fig. 3A. The PE insert thickness influenced when the post engaged with the cam, which occurred after 60° in the overstuffed configurations, after 60°–75° in the reference configurations and after 75° in the understuffed configurations. In the overstuffed configurations, there were no differences between KA and MA. In the reference and understuffed configurations, MA caused contact locations that were initially anterior to the KA configurations but at the 90° flexion they were all at the same position.

The axial rotation kinematics are shown in Fig. 3B. At full extension, all configurations were internally rotated relative to their position at 90°. Increasing the PE insert thickness increased the internal rotation for each alignment. However, in flexion of 75°–90°, all those in MA had decreased internal rotation compared to those in KA regardless of the PE insert thickness. During 15°–75° of neutral flexion the two alignments yielded different waveforms.

The coronal kinematics are shown in Fig. 3C. In the first 30° of knee flexion all the configurations were in a varus alignment but by 30° of knee flexion all demonstrated a neutral coronal alignment. This degree of initial varus was influenced by the PE insert thickness and associated ligament tension. Increasing PE insert thickness resulted in decreasing initial varus as the sMCL tension increased more compared to the LCL.

### Laxity testing

**Fig. 4:**
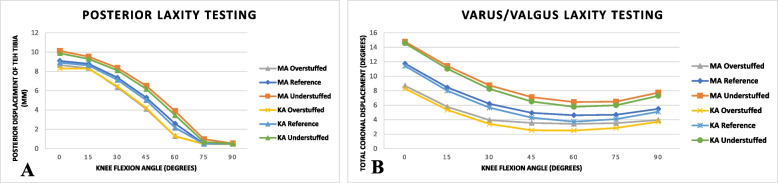
Graphs illustrating the joint laxity test results. **A** Posterior, **B** Varus/valgus

The posterior laxity is shown in Fig. 4A. The posterior laxity is greatest at full extension and decreases as the knee flexion increases. After the post-cam mechanism engages, the observed posterior laxity becomes negligible due to the mechanical block of the post on the cam. The PE insert thickness influences the laxity; a 2 mm change of PE inserts in the configurations resulted in a mean laxity difference of 0.7 mm (14%) and 0.6 mm (12%) in MA and KA models respectively. This difference in laxity increased with understuffing and decreased with overstuffing of the joint space. This corresponds to previously observed ligament tension changes with PE insert thickness during neutral flexion Fig. 2.

The VV laxity testing results are shown in Fig. 4B. The coronal constraint is positively influenced by the PE thickness with increased thickness demonstrating less coronal laxity. The understuffed configurations compared to the reference configurations resulted in a mean 2.0° (28%) and 2.0° (31%) reduction in the coronal laxity in MA and KA respectively. The least coronal laxity is observed at 60° of knee flexion which coincides with peak sMCL tensions, and the LCL tension is at its peak at 75° of knee flexion.

### Joint compressive forces

**Fig. 5:**
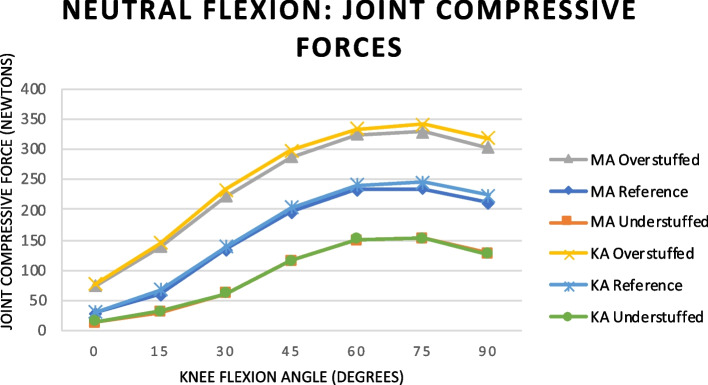
Graph illustrating the joint compressive forces during neutral flexion

The joint compressive forces (JCFs) results are shown in Fig. 5. The under- and overstuffed configurations influenced the JCFs. The overstuffed compared to the reference configuration resulted in a mean JCFs increase by 73 N (61%) and 77 N (62%) in MA and KA models respectively. The inverse was yielded with the understuffed configuration. The peak forces are recorded from 60°–75° of knee flexion peaking at 75°. The peak forces in the overstuffed configurations were 329 N and 340 N in MA and KA respectively. The peak forces in the understuffed configurations were 152 N and 155 N in MA and KA respectively. The peak forces in the reference configurations were 235 N and 245 N in MA and KA respectively. This coincidence with the peak collateral ligament tension is shown in Fig. 2, the engagement of the post-cam mechanism Fig. 3A and the least coronal laxity in Fig. 4B. This illustrates the positive relationship between ligament tension, stability and JCFs.

## Discussion

The goal of our study was to describe the effect of under- and overstuffing the joint space in PS-TKA on knee kinematics, joint forces and laxity. Using MA and KA, we measured and recorded data during neutral flexion and laxity testing. To our knowledge, this is the first study to evaluate the effects of under- and overstuffing the tibiofemoral joint, in both KA and MA, in relation to neutral flexion, laxity, JCFs, and post-cam engagement in PS-TKA.

In the PS-TKA, the function of the PCL is substituted by the post-cam mechanism. The post-cam mechanism engages at around 75° of flexion, this engagement prevents anterior femoral translation of the femur and facilitates the femoral rollback in PS-TKA [31, 32]. PS-TKA has been shown to increase the sagittal constraint of the implanted knee [33]. Hamai *et al.* [34] evaluated CR- and PS-TKA *in vivo* kinematics using radiographically-based image-matching techniques. They found the post-cam mechanism did not engage during stair climbing but this was due to the dynamic flexion angle in their study being less than 75°. They found that CR-TKA demonstrated more sagittal stability. Several studies have evaluated the post-cam mechanism under the following areas of interest: geometry, wear, contact forces, kinematics and design [35–40]. Our study suggests that increasing the PE insert thickness during may confer greater sagittal stability during activities of daily living, such as stair climbing. This is based on our observation that, with increased PE insert thickness and overstuffing, the joint space resulted in the post-cam mechanism engaging earlier after 60° of flexion demonstrated in our neutral flexion AP translation kinematics in the overstuffed configurations (Fig. 3A). The observations in our study are likely driven by the ligament tension increases yielded in the overstuffed configurations.

We observed a positive relationship between increasing PE insert thickness (overstuffing the joint space) and ligament tension (Fig. 2). Whilst observing increasing PE insert thickness occurs with decreased joint laxity (Fig. 4). Shimizu *et al.* [41] investigated the effect of weight bearing on PS-TKA kinematics. They reported post-cam engagement at 93.4° ± 3.3° and 70.5° ± 7.2° in weight-bearing and non-weight bearing respectively. In loading the joint, the ligaments become lax,  resulting in later engagement of the post-cam. This phenomenon of later post-cam engagement with laxity was observed in our understuffed configurations (Fig. 3A). It has been suggested that delayed post-cam engagement can facilitate increased maximum knee flexion [42, 43]. Suggs *et al.* [43] showed a correlation between the initial post-cam contact angle and the maximum flexion angle *r* = 0.505 (*P* = 0.019). Arnout *et al.* [5] demonstrated in their in vitro study using a dynamic knee kinematic simulator that earlier post-cam engagement facilitated more physiological motion. The post-cam mechanism determines the posterior femoral translation and facilities movement in deeper flexion shown in a computation model study [44]. We observed that understuffing the joint space resulted in increased joint laxity with delayed post-cam engagement. Overall, this offers a biomechanical explanation of observations in the clinical literature that report increased ROM with increased joint laxity [45].

There are studies that have shown that early post-cam engagement can lead to increased contact stress and accelerate post-wear [46, 47]. However, these effects are further highlighted in our posterior laxity testing shown in Fig. 4A. This is driven by the related JCFs. As the post-cam mechanism engages, the observed posterior laxity becomes negligible due to the mechanical block of the post on the cam. The posterior laxity yielded in our study at full extension when the native knee is most stable, is likely a result of the lack of secondary knee stabiliszers in our virtual model. A radio stereometric analysis study demonstrated that kinematics in PS-TKA can impact tibial component migration through alterations in the force transmission [48]. The kinematic differences we observed were coupled with changes in the JCFs (Fig. 5). These subtle variations in JCFs may impact long-term implant survivorship. Further studies are required to evaluate how the JCFs changes we observed translate into contact stress patterns.

A manufacturer indicated their PS-TKA designs allow for up to ±12° of axial rotation [49]. Cates *et al.* [50] evaluated the *in vivo* kinematics using fluoroscopy and reported in PS-TKA that from full extension to 90° flexion internal rotation of the tibia. The axial rotation means angles at full extension were –1.0° in PS-TKA, and at maximum flexion, the mean axial rotation angle was 1.9° for PS-TKA. Tamaki *et al.* [51] evaluated the *in vivo* kinematics of 20 TKA and reported the femoral component demonstrated a mean of 13.5° (5.2° to 21°) external rotation of increasing external rotation with flexion. We observed similar axial rotation, *i.e.*, tibial internal rotation in the first 30° and 45° in MA and KA configurations respectively. For native knee kinematics, in the initial 30° of flexion, the tibial internally rotates thereafter it remains within 1° of that position while PS-TKA typically continues to internally rotate [52]. Our configurations demonstrated reduced internal rotation with continued flexion. This observation isn't abnormal for the implanted knee as Suggs *et al.* [43] reported that with post-cam engagement there is a reduction in tibial internal rotation. The coronal laxity decreased in our study by overstuffing the joint. This can be explained by the increased ligament tension which increases the stability of the configurations.

Our study findings suggest that TKA alignment and not PE insert thickness may play a greater role in axial rotation in PS-TKA with greater internal rotation in KA compared to MA irrespective of the PE insert thickness (Fig. 3B). The internal rotation of the tibia with flexion is related to the screw-home mechanism observed in the knee. The greater internal rotation observed in KA is in keeping with literature which supports KA as being more physiological [53, 54]. Another in silico study demonstrated more physiological knee kinematics with KA [55]. Amongst the two alignments, KA allows a greater contribution of the soft tissues to balance and stabilize the knee. The increased contribution of the soft tissues is evidenced by the increased JCF and soft tissue tension proportionately in KA compared to MA. Our study yielded higher JCFs with KA (Fig. 4). High contact pressures have been reported in KA, which may result in increased PE wear but have not been shown to cause PE failure [55]. Our study demonstrated minor differences between the alignments with regards to the joint kinematics during neutral flexion, laxity testing in conjunction with under- and overstuffing the tibiofemoral joint. This is representative of the current literature regarding alignment which remains controversial [53, 54, 56, 57].

The limitations of this study were the use of point-to-point ligaments rather than bundles of ligaments which do not completely represent the native ligament properties. There is still a significant variation in the literature regarding the representation of the ligaments [30]. The computational models are based on approximations and assumptions made to simplify the complexity of the human knee [58]. Our model lacked the patellofemoral joint and therefore its effect on TKA kinematics was excluded in our study.

## Conclusion

Surgeons who utilise PS-TKA need to be aware that PE size changes have a variable effect on the kinematics of the implanted knee. These effects are in conjunction with other features such as alignment and the post-cam mechanism. This body of work contributes to the understanding of TKA kinematics in PS-TKA. This knowledge can aid the surgeon in intraoperative decision-making to individualize patient treatment. Improvement in judgement can improve patient satisfaction and mitigate against pain, instability and subsequent revisions while optimizing kinematics.

## Data Availability

Yes.

## Abbreviations

6 DoF: 6 degrees of freedom
AP: Anteroposterior
CR: Cruciate-retaining
IE: Internal/external rotation
JCFs: Joint compressive forces
KA: Kinematic alignment
LCL: Lateral collateral ligament
MA: Mechanical alignment
PCL: Posterior cruciate ligament
PE: Polyethylene
PS: Posterior-stabilized
ROM: Range of motion
sMCL: Superficial medical collateral ligament
TKA: Total knee arthroplasty
VV: Varus/valgus
